# Airy beam optical parametric oscillator

**DOI:** 10.1038/srep25245

**Published:** 2016-05-04

**Authors:** A. Aadhi, N. Apurv Chaitanya, M. V. Jabir, Pravin Vaity, R. P. Singh, G. K. Samanta

**Affiliations:** 1 Photonic Sciences Lab., Physical Research Laboratory, Navarangpura, Ahmedabad 380009, Gujarat, India; 2 Indian Institute of Technology-Gandhinagar, Ahmedabad 382424, Gujarat, India

## Abstract

Airy beam, a non-diffracting waveform, has peculiar properties of self-healing and self-acceleration. Due to such unique properties, the Airy beam finds many applications including curved plasma wave-guiding, micro-particle manipulation, optically mediated particle clearing, long distance communication, and nonlinear frequency conversion. However, many of these applications including laser machining of curved structures, generation of curved plasma channels, guiding of electric discharges in a curved path, study of nonlinear propagation dynamics, and nonlinear interaction demand Airy beam with high power, energy, and wavelength tunability. Till date, none of the Airy beam sources have all these features in a single device. Here, we report a new class of coherent sources based on cubic phase modulation of a singly-resonant optical parametric oscillator (OPO), producing high-power, continuous-wave (cw), tunable radiation in 2-D Airy intensity profile existing over a length >2 m. Based on a MgO-doped periodically poled LiNbO_3_ crystal pumped at 1064 nm, the Airy beam OPO produces output power more than 8 W, and wavelength tunability across 1.51–1.97 μm. This demonstration gives new direction for the development of sources of arbitrary structured beams at any wavelength, power, and energy in all time scales (cw to femtosecond).

In the context of quantum mechanics, Berry and Balazs^1^ in 1979 theoretically predicted the existence of a unique wave packet solution of a free particle Schördinger equation in the form of a non-spreading Airy function. The most striking features of such wave packet is its free space propagation in a parabolic trajectory (*free acceleration*) even in the absence of any external potential. The *free acceleration* of the Airy beam does not contradict with Ehrenfest’s theorem describing the motion of the centre of gravity of a wave packet^2^ as the Airy beam acceleration is attributed to its caustic of the wave packet. Mathematical resemblance between the free particle Schrödinger equation and the paraxial Helmholtz equation has enabled theoretical^3^ and experimental^4^ realization of finite energy Airy beam in the field of optics. Airy beam has attracted a great deal of interest in understanding its ballistic propagation dynamics^5^,^6^,^7^,^8^, along with invariant intensity profile (non-diffraction) and self-restoration of its canonical form (self-healing) even after obstruction by small objects^9^ as well as its interesting applications in basic science and technology^10^,^11^,^12^,^13^,^14^,^15^,^16^,^17^,^18^,^19^. The experimental generation of Airy beam relies on Fourier transformation (FT) of a cubic phase modulated Gaussian beam. To date, the Airy beams generated through different techniques^4^,^19^ suffer from common drawbacks of low power, energy, and limited or no wavelength tunability. Efforts have been made for compact Airy beam laser^20^,^21^, with low output power and fixed wavelength.

Optical parametric oscillators (OPOs)^22^,^23^,^24^ especially in singly-resonant (SROs) configuration offer the most viable solution for high-power radiation over extended spectral regions inaccessible to lasers. Unlike lasers, continuous-wave (cw) SROs have been traditionally the most challenging class of devices due to substantially lower nonlinear gains available under cw pumping, resulting in high operation threshold. Recent advancement in fiber laser technology and the availability of improved periodically-poled crystals have displayed the SRO operation even with substantial cavity losses^23^. Therefore, direct generation of Airy beam from the SROs can be a major step for the realization of a compact high-power source of structured spatial beams with wide wavelength tunability. Here, we report, for the first time to the best of our knowledge, a source of high-power, cw, tunable radiation in 2-D Airy intensity profile based on cubic phase modulation of a cw SRO using an intra-cavity diffractive optical element. The source provides Airy beam with power as high as 8 W, and wavelength tuning across 1.51–1.97 μm. This is a generic approach, which in principle can be extended to any wavelength across the electromagnetic spectrum in all time scales (cw to femtosecond) with any structured beam.

## Experiment

**Figure 1:**
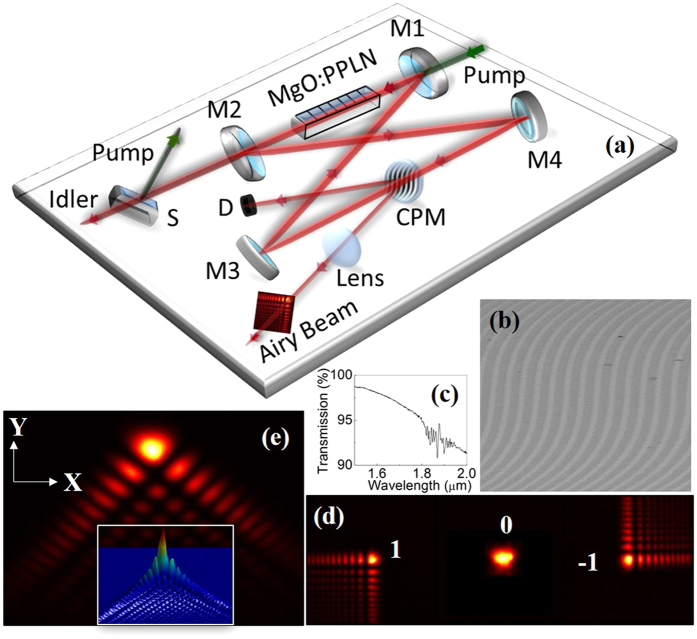
Airy beam optical parametric oscillator. (**a**) Schematic of the experimental setup. M1–4; mirrors, MgO:PPLN; nonlinear crystal for OPO, CPM; cubic phase mask, S; separator, (**b**) Microscopic image of the CPM, (**c**) Transmission of CPM for 0^th^ order beam, (**d**) Diffraction pattern of the CPM, (**e**) 2D and 3D intensity profile of the Airy beam at Fourier plane.

In the experiment, we have devised the SRO in a compact four mirror ring cavity (Fig. 1(a), and see Methods) consisting of two curved mirrors, M1 and M2 and two plane mirrors, M3 and M4. A 50 W cw Yb-fiber laser at 1.064 μm is used to pump a multi-grating MgO:PPLN crystal to produce signal and corresponding idler radiation across 1.45–2 μm and 2.1–5 μm respectively. All the cavity mirrors are having high reflectance for signal wavelengths and high transmittance for pump and idler to ensure singly resonant condition. To generate finite energy Airy beam, we designed a cubic phase mask (CPM) (Fig. 1(b)) in the form of a binary diffraction grating which modulates both the phase and amplitude of the diffracted beam^21^. The profile of the binary diffraction grating can be represented as^21^,^25^


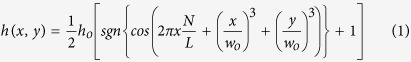


where, *w*_*o*_ is a constant. The period of the grating determined by the ratio of *N*, the number of lines of the grating and *L*, length of the grating in *x* direction. The normalized complex amplitude of the 1^st^ order diffracted beam in the presence of cubic phase modulation carries phase given by,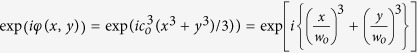. The constant, 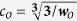 of the CPM determines the cubic phase imposed to the input beam. In current experiment, the grating have total *N* = 100 lines over width of *L* = 2 mm and *w*_*o*_ = 250 μm. The value of *c*_*o*_ is estimated to be 5.77/mm. The ridge height, *h*_*o*_ is optimized to have zero-order transmission and diffraction efficiency of first-order (+1 and −1) diffracted beams at wavelength of 1.6 μm of *T* = 98% and *η*_*D*_~2% respectively. It has varying zero-order transmission from 98.7% to 91.3% across 1.5–2 μm (Fig. 1(c)). The binary grating has aperture of 2 × 2 mm^2^ on a fused silica plate and carrier period of 20 μm. The +1 order diffracted beam of the CPM placed inside cavity between mirrors M3 and M4 is Fourier transformed into Airy beam using a lens of focal length *f* = 300 mm. Figure 1(e) shows 2D and 3D intensity distribution of the Airy beam.

## Discussions

**Figure 2:**
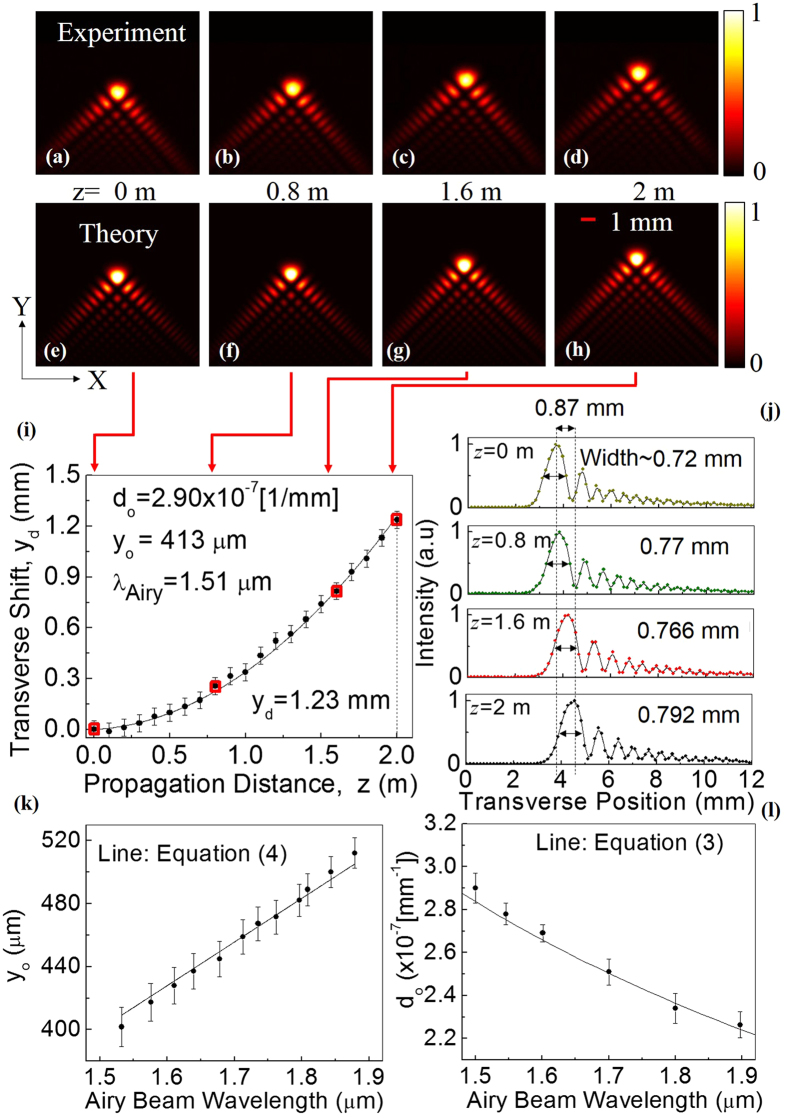
Propagation dynamics of the Airy beam. (**a**–**d**) Experimental, and (**e**–**h**) simulated 2D intensity distribution of the Airy beam of wavelength 1.51 μm along the propagation at z = 0, 0.8, 1.6 and 2 m. (**i**) Experimental (dots) trajectory of the 2D Airy beam along with theoretical fit (equation (2)). The error bar corresponds to pixel size (±42.5 μm). (**j**) 1D line profile of the Airy beam along propagation showing non-divergence. (**k**,**l**) Dependence of characteristic length, *y*_*o*_ and deflection coefficient, *d*_*o*_ of Airy beam on its wavelength respectively. Solid lines are theoretical fit of equation (3) and equation (4) to the experimentally measured characteristic length, *y*_*o*_ and deflection coefficient, *d*_*o*_respectively. Error bars in plots k and l are fractional and fitting errors respectively.

To verify the generation of Airy beam, we explored the self-acceleration, non-diffraction and self-healing properties. Operating the SRO at an arbitrary signal wavelength (say 1.51 μm) across its tuning range (see Methods) we have recorded 2-D intensity distribution of the +1 order diffracted beam of the phase grating along the propagation direction with z = 0 as the Fourier plane of the lens using a large area (12 × 12 mm^2^) pyro-electric array camera of pixel size 85 × 85 μm^2^. The first row, (a–d) and the second row, (e–h) of Fig. 2 show the experimental and numerical (simulated using theoretical expressions for propagation dynamics) transverse profiles respectively at propagation distances, z = 0, 0.8, 1.6 and 2 m. As evident from Fig. 2(a–d), the Airy beam shows transverse shift (see Y-direction of the images) from the rectilinear propagation. The numerical transverse profiles (Fig. 2(e–h)) calculated using the experimental parameters, show close agreement with the experimental results.

To get quantitative understanding about the acceleration we have recorded the intensity profile of the Airy beam at different propagation distances and measured the position of the central lobe of the Airy profile with the results shown in Fig. 2(i). As evident from Fig. 2(i), the Airy beam moves away from its position at z = 0 quadratically with propagation distance, z, resulting a shift of *y*_*d*_ = 1.23 mm at z = 2 m. The symmetric 2D Airy beam of wavelength, λ incident on the FT lens at a launching angle, *θ*, have the ballistic dynamics with the transverse shift as^26^,^27^,


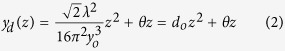


The deflection coefficient, *d*_o_ and characteristic length, *y*_*o*_ are given as^27^,


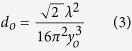


and


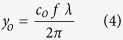


Here, *f* is the focal length of the FT lens and *c*_*o*_ is the constant determined by the CPM parameters.

We fit the experimental results (Fig. 2(i)) with a quadratic equation (correlation coefficient of fitting, r^2^ ~0.99), compared the coefficients with that of equation (2) and found the values of *y*_*o*_ and *θ* to be 413 μm and ~40 μrad, respectively. Using the parameters, *c*_*o*_ = 5.77/mm, *f* = 300 mm and λ = 1.51 μm in equation (4) we calculate *y*_*o*_ to be ~416 μm, which is very close to the experimental value of *y*_*o*_ (~413 μm). To verify the non-divergence we have recorded the 1D line profile of the Airy beam at z = 0, 0.8, 1.6 and 2 m with the results shown in Fig. 2(j). As evident from Fig 2(j), the width (full width at half maxima, FWHM) of the central lobe of the Airy beam measured from Gaussian fit varies from 0.720 ± 0.06 mm to 0.792 ± 0.06 mm for the beam propagation from z = 0 to z = 2 m. The increase in the beam width is comparable to the width of the pixel (85 μm) proving the non-divergence of the Airy beam even after a propagation distance of 2 m. The shift in the position of the central lobe of the Airy beam is attributed to the beam acceleration. Similar behaviour is observed for Airy beam of different wavelengths across the tuning range of the SRO.

From equation (3) and equation (4) it is evident that for a fixed FT lens and CPM, *y*_*o*_ and *d*_*o*_parameters of the Airy beam are respectively proportional and inversely proportional to its wavelength. To verify the dependence of *y*_*o*_ and *d*_*o*_ parameters on wavelength we have recorded the Airy intensity distribution and transverse displacement of the beam with propagation distance for different wavelengths. The *y*_*o*_ value measured by direct fitting of Airy function to the transverse profile of the Airy beam^27^ show linear dependence with its wavelength (Fig. 2(k)). Using experimental values of *c*_*o*_ = 5.77/mm and *f* = 300 mm, we find a close agreement of equation (4) (solid line) to the experimental data (dots). Therefore, it is trivial to predict from equations (3) and (4) that the deflection coefficient *d*_*o*_ is inversely proportional to wavelength. However, *d*_*o*_ value measured by fitting equation (2) to the beam deflection with propagation at different wavelengths also prove inverse relation of the deflection coefficient (acceleration) of the Airy beam to its wavelength (Fig. 2(l)). Such finding validates that the Airy beam OPO provides output beam with tunable acceleration.

**Figure 3:**
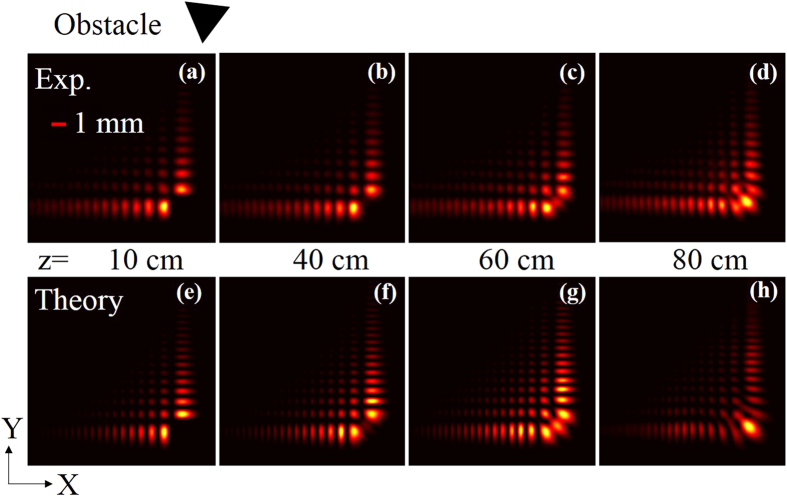
Self-healing property of the Airy beam. (**a–d**) Experimental profile photograph verses numerical simulation, (**e**–**h**), at propagation distances, z = 10, 40, 60 and 80 cm after the Fourier plane (z = 0). The central lobe of the Airy beam is blocked using a knife edge (obstacle).

To verify the self-healing behaviour, we blocked the central lobe of the Airy beam using a triangular obstacle (knife edge, see Fig. 3) at the Fourier plane and recorded the intensity distribution of the beam at different distances, z = 10 cm, 40 cm, 60 cm and 80 cm with the results shown in Fig. 3. As evident from Fig. 3 (a), the Airy beam has no central lobe at z = 10 cm, however, during propagation, the beam shows sign of healing at a distance ~60 cm (Fig. 3(c)) with almost complete regeneration at a distance of ~80 cm (Fig. 3(d)). The beam maintains same intensity distribution in the course of further propagation. Using experimental parameters (wavelength and *y*_*o*_) we simulated (using numerical model, see methods) Airy beam intensity profile for self-healing study along propagation (Fig. 3(e–h)) which is in good agreement with the experimental results.

**Figure 4:**
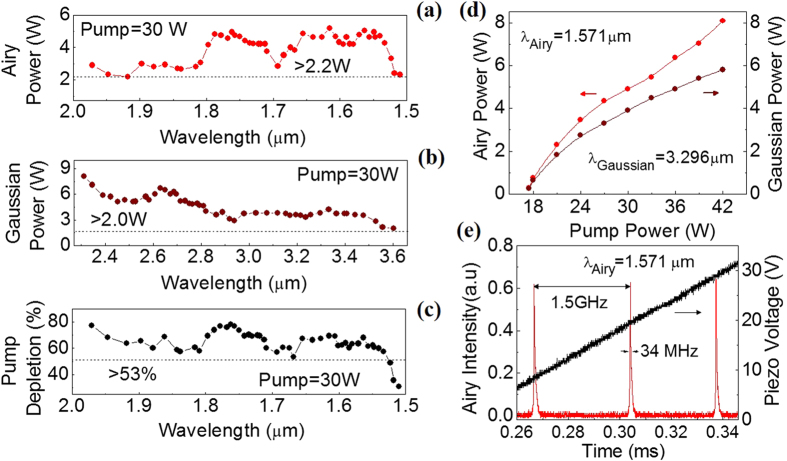
Performance parameters of the Airy beam source. (**a**) Airy beam power across the tuning range. (**b**) The Gaussian beam power across the mid-IR wavelength range. (**c**) Variation of pump depletion of the Airy beam source. (**d**) Power scaling of the Airy beam source. Lines are guide to eyes. (**e**) Single frequency nature of the Airy beam radiation measured using a scanning Fabry-Perot interferometer.

Subsequently, we have studied the performance of the Airy beam source in terms of power scaling property, power across the tuning range and its single-frequency nature with the results shown in Fig. 4. To measure the output power of the Airy beam across the tuning range we pumped the OPO at a constant power (30 W) and recorded the Airy beam power while tuning its wavelength across 1.51–1.97 μm through the variation of crystal temperature and grating period over 35 –200 °C and 30.0–31.5 μm respectively, with the results shown in Fig. 4(a). The Airy beam power, as evident from Fig. 4(a), varies from 2.3 W at 1.51 μm to 2.9 W at 1.97 μm with a maximum of 5.18 W at 1.61 μm with extraction efficiency of 17.2%. Across entire wavelength range the Airy beam power is >2 W. In addition, the Airy beam source produces coherent radiation in the mid-IR wavelength range across 3.6–2.31 μm in Gaussian spatial distribution with maximum power of 8 W at 2.31 μm (Fig. 4(b)). The source has pump depletion of ~80% near degeneracy, however, reduces to 31% for the Airy beam source at 1.51 μm. The pump depletion of the source is >53% across the tuning range (Fig. 4(c)). To study the power scaling property of the Airy beam OPO we have measured the variation of Airy beam power at wavelength 1.57 μm with the pump power. The results are shown in Fig. 4(d). The output power of the Airy beam (red dot and line) increases with the pump power from an operation threshold of 17.5 W producing a maximum power of 8.1 W for 42 W of pump power at 19% extraction efficiency. Similarly, the output power of the Gaussian beam (brown dot and line) at 3.30 μm increases with the pump. No sign of saturation indicates the possibility of further enhancement in Airy beam power with increase of pump power. Under this operating condition, the Airy beam source has pump depletion >70%. We also verified the single frequency nature of the Airy beam source using a scanning Fabry-Perot interferometer^28^ (Free spectral range, FSR = 1.5 GHZ, Finnese 200) with the results shown in Fig. 4(e). The instantaneous line-width of the transmission peaks can be estimated to be ~34 MHz.

In conclusion, we have extended the OPOs commonly producing tunable optical radiation in Gaussian spatial intensity distribution, for the first time, to generate optical radiation in Airy intensity distribution. Our method allows transfer of all inherent advantages of the OPOs in terms of wide wavelength tunability across the electromagnetic spectrum, high power and energy in all times scales (cw to ultrafast) to the Airy beam. The wide wavelength coverage of the Airy beam source, has enabled us to verify the dependence of Airy beam parameters to its wavelength. On the other hand, the generation of such beam with high power enables the possibility of using Airy beam source in many fields including nonlinear interaction with matter. The concept is generic and can be used to generate any structured beam.

## Methods

### Experimental setup

The schematic of the experimental setup of Airy beam SRO is shown in Fig. 1(a). A cw fiber laser (IPG, USA) of 50 W output power at 1.064 μm is used to pump the SRO. The SRO is designed in a compact four mirror ring cavity^28^ consisting of two curved mirrors, M1 and M2 with radius of curvature, *r* = 100 mm, and two plane mirrors M3 and M4. All the OPO mirrors are having high reflectance (R>99%) for signal radiation across 1.45–2 μm and high transmittance (T >80%) for pump and idler radiation over 2.1–5 μm ensuring singly resonant condition. A 50-mm long and 8.6 × 1 mm^2^ in aperture, multi-grating MgO:PPLN nonlinear crystal (HC Photonics), with grating periods varying from Λ = 28.5–31.5 μm in 0.5 μm steps, housed in an oven is used for SRO. The oven temperature can be varied from 30–200 °C with temperature stability of ±0.1 °C. A lens (not shown in Fig. 1(a)) of *f* = 100 mm is used to focus the pump laser to a beam waist diameter of ~79 μm at the centre of the nonlinear crystal. A transmission based diffraction cubic phase mask (CPM) of 2 × 2 mm^2^ fabricated at the centre of a fused silica plate of diameter Φ = 25.4 mm and thickness of 3 mm is placed in between mirrors M3 and M4 for phase modulation of the resonant signal beam. The grating has N = 100 lines in L = 2 mm resulting a carrier period of 20 μm. The phase mask has 0^th^ order transmission of ~98% at 1.6 μm resulting ~2% coupling of the intra-cavity signal into Airy beam. To test the diffraction of the CPM, we incident a Gaussian beam of wavelength 1.6 μm and recorded the diffracting orders +1 and −1 with cubic phase modulated beams and the 0^th^ order high intense non-diffracted Gaussian beam (Fig. 1(d)). The 0^th^ order beam was attenuated while observing the diffracted beams. We did not observe higher order diffracted beams with substantial power. Using ABCD matrix formalism we have designed the SRO cavity. The estimated beam radius at the phase mask is ~700 μm. The total length of the cavity is 76 cm. A lens of focal length *f* = 300 mm is used for the Fourier transformation.

### Recording of propagation dynamics of Airy beam and temperature tuning of the SRO

To avoid the use of any reference beam to measure the deflection of the Airy beam from the straight line path, we have rotated the cubic phase mask as compared to its conventional orientation^4^ by 45^o^ counter clockwise resulting Airy beam acceleration in the direction perpendicular (Y-direction) to the optical table. As a result, the deviation of the beam height along the propagation distance z from the beam height at z = 0 gives direct measure of Airy beam acceleration. A large area (12.5 mm × 12.5 mm) pyro-electric array camera (Pyrocam-III-C-A, Ophir, USA), of pixel size 85 μm × 85 μm is used to record the Airy beam intensity profile along the propagation. From the images we have derived the intensity profile of Airy beam. These profiles were used to derive the results of Fig. 2 and Fig. 3. The centre of the main lobe of Airy beam, required for acceleration study in Fig. 2(i), is found by fitting to the experimental images. The error bar, ±42.5 μm, in Fig. 2(i), corresponds to pixel size. The deflection coefficient, *d*_*o*_ is estimated by fitting equation (2) to the experimentally measured transverse shift of the Airy beam. The error bars in Fig. 2(l) are the fit error to find deflection coefficients. The characteristic length, *y*_*o*,_ of the Airy beam is found by fitting theoretical expression of 1D Airy beam to 1D line profile of the Airy beam and measuring the distance between the central and the first maxima^27^. The error bars in Fig. 2(k) are the fractional errors. The aperture parameter, *a*_*o*_, of the Airy beam can be calculated from the exponential decay of the Airy wave function.

### Numerical simulation

We have simulated self-healing property of Airy beam by solving paraxial Helmholz equation numerically using split-step Fourier transform method^29^. We have considered the complex field amplitude of Airy beam at z = 0, multiplied it with a square aperture function numerically to block the central lobe and propagated the field using beam propagation method^29^.

## Additional Information

**How to cite this article**: Aadhi, A. *et al.* Airy beam optical parametric oscillator. *Sci. Rep.* **6**, 25245; doi: 10.1038/srep25245 (2016).
